# Comprehensive bioinformatics analysis of ribonucleoside diphosphate reductase subunit M2(RRM2) gene correlates with prognosis and tumor immunotherapy in pan-cancer

**DOI:** 10.18632/aging.204315

**Published:** 2022-10-03

**Authors:** Liyuan Wu, Le Yin, Linxiang Ma, Jiarui Yang, Feiya Yang, Baofa Sun, Xing Nianzeng

**Affiliations:** 1 Department of Urology, National Cancer Center/National Clinical Research Center for Cancer, Cancer Hospital, Chinese Academy of Medical Sciences and Peking Union Medical College, Beijing 100021, China; 2 State Key Laboratory of Molecular Oncology, National Cancer Center/National Clinical Research Center for Cancer/Cancer Hospital, Chinese Academy of Medical Sciences and Peking Union Medical College, Beijing 100021, China; 3 Research and Development Department, Allife Medicine Inc., Beijing 100176, China; 4 Department of Urology, Weifang Hospital of Traditional Chinese Medicine, Weifang 261000, Shandong, China; 5 Tianjin Key Laboratory of Animal and Plant Resistance, College of Life Sciences, Tianjin Normal University, Xiqing, Tianjin 300382, China; 6 Department of Zoology, College of Life Science, Nankai University, Nankai, Tianjin 300071, China; 7 Department of Urology, Shanxi Province Cancer Hospital/Shanxi Hospital Affiliated to Cancer Hospital, Chinese Academy of Medical Sciences/Cancer Hospital Affiliated to Shanxi Medical University, Shanxi, Taiyuan 030013, China

**Keywords:** ribonucleotide reductases, ribonucleotide-diphosphate reductase subunit M2, pan-cancer, bioinformatic analyses, immunotherapy

## Abstract

Ribonucleotide reductase (RNR) small subunit M2 (RRM2) levels are known to regulate the activity of RNR, a rate-limiting enzyme in the synthesis of deoxyribonucleotide triphosphates (dNTPs) and essential for both DNA replication and repair. The high expression of RRM2 enhances the proliferation of cancer cells, thereby implicating its role as an anti-cancer agent. However, little research has been performed on its role in the prognosis of different types of cancers. This pan-cancer study aimed to evaluate the effect of high expression of RRM2 the tumor prognosis based on clinical information collected from The Cancer Genome Atlas (TCGA) and The Genotype-Tissue Expression (GTEx) databases. We found RRM2 gene was highly expressed in 30 types of cancers. And we performed a pan-cancer analysis of the genetic alteration status and methylation of RRM2. Results indicated that RRM2 existed hypermethylation, associated with m6A, m1A, and m5C related genes. Subsequently, we explored the microRNAs (miRNA), long non-coding RNAs (lncRNA), and the transcription factors responsible for the high expression of RRM2 in cancer cells. Results indicated that has-miR-125b-5p and has-miR-30a-5p regulated the expression of RRM2 along with transcription factors, such as CBFB, E2F1, and FOXM. Besides, we established the competing endogenous RNA (ceRNA) diagram of lncRNAs-miRNAs-circular RNAs (circRNA) involved in the regulation of RRM2 expression. Meanwhile, our study demonstrated that high-RRM2 levels correlated with patients’ worse prognosis survival and immunotherapy effects through the consensus clustering and risk scores analysis. Finally, we found RRM2 regulated the resistance of immune checkpoint inhibitors through the PI3K-AKT single pathways. Collectively, our findings elucidated that high expression of RRM2 correlates with prognosis and tumor immunotherapy in pan-cancer. Moreover, these findings may provide insights for further investigation of the RRM2 gene as a biomarker in predicting immunotherapy’s response and therapeutic target.

## INTRODUCTION

Ribonucleotide reductase (RNR) is responsible for the de novo synthesis of deoxyribonucleotide triphosphate (dNTP), thereby playing an important role in DNA synthesis and DNA repair. However, abnormal dNTP levels lead to inaccurate DNA replication, causing genomic instability [1]. The activity of RNR is coordinated with the cell cycle and the levels of its smaller subunit ribonucleotide reductase subunit M2 (RRM2). Apart from the catalytic RRM2, the enzyme RNR is made up of a regulatory larger subunit, i.e., ribonucleotide reductase subunit M1 (RRM1) [2]. In humans, RNR comprises one large subunit RRM1 and two small subunits, RRM2B and RRM2. The expression of RRM1 remains the same throughout the cell replication cycle, but the expression of RRM2 increases during the early S phase and the late G1 phase [3]. During the G2 phase, following cyclin-dependent kinases (CDK)-mediated phosphorylation of Thr33, RRM2 is degraded via cyclin F to maintain balanced levels of dNTPs [4]. Therefore, the level of RRM2 regulates the cell cycle-dependent activity of RNR, the rate-limiting enzyme.

Expression levels of RNR subunits have been studied in various types of cancers, leading to the findings of over-expression of RRM2 in cancer cells [5]. The higher expression of RRM2 has also been associated with poor survival outcomes in cancer patients [6]. Because of these functions, RNR inhibitors have been widely utilized in cancer treatment along with chemotherapy [7, 8]. Studies have indicated that high-expression levels of RRM2 can be regulated at non-coding RNAs (ncRNAs) and RNA modification [6, 9]. Besides, some researchers reported that RRM2 could regulate immunotherapy responses, and the knockdown of RRM2 could enhance the anti-tumor efficiency of PD-1 blockade in renal carcinoma [10]. Therefore, all findings suggest that RRM2 plays an important role in tumorigenesis, tumor progression, and the treatment of cancer.

The public database The Cancer Genome Atlas (TCGA) project contains functional genomics datasets, thereby providing us with high-throughput RRM2 expression data and clinical information of cancer patients. Therefore, in this study, we utilized the TCGA data to establish a comprehensive bioinformatics-based pan-cancer analysis of RRM2. Besides, we performed analyses to explore the potential mechanism responsible for the pathogenesis. The overall study flowchart is shown in Supplementary Figure 1A.

## RESULTS

### RRM2 expression and survival analyses in human cancers

**Figure 1:**
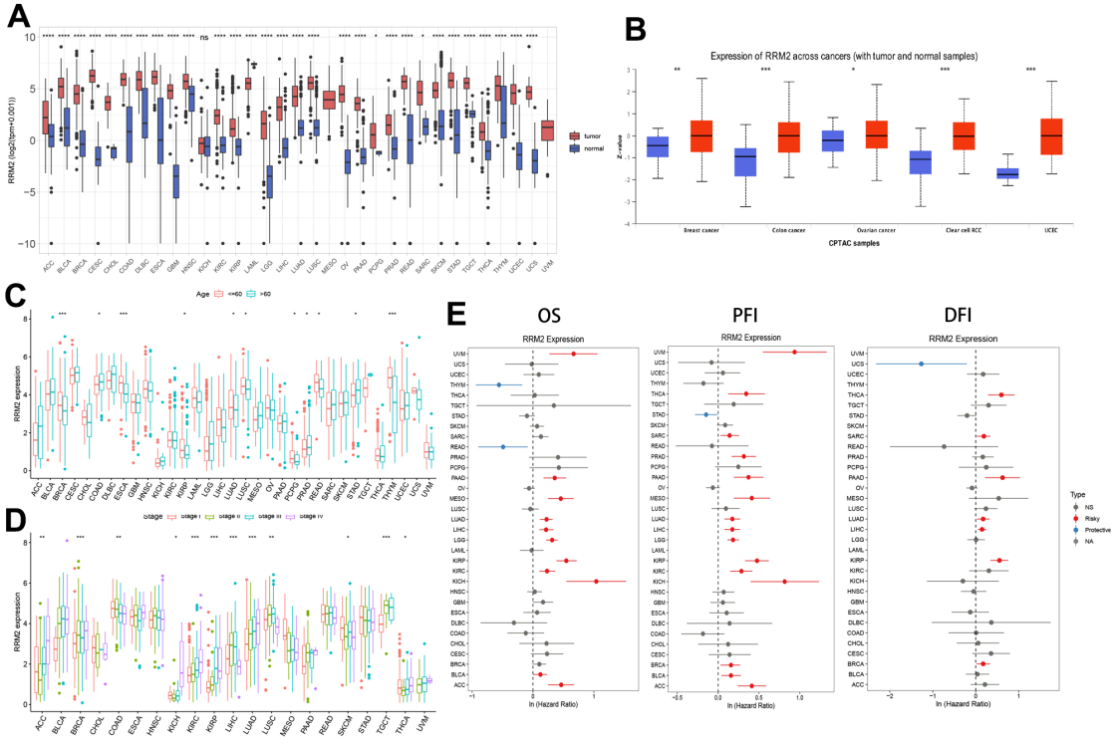
***RRM2* gene expression levels in different tumors and clinical data of cancer patients obtained from TCGA.** (**A**) *RRM2* gene expression levels in different cancer types and normal tissue from the TCGA and GTEx datasets. The red rectangle box represents the gene expression level in tumor tissue and the blue rectangle box in normal tissue. (**B**) Protein expression of RRM2 in breast cancer, ovarian cancer, colon cancer, clear cell RCC, and UCEC. The red rectangle box represents protein expression levels in tumor tissue and the blue one in normal tissue. (**C**) The *RRM2* gene expression levels in patients belonging to different age groups. The red rectangle box indicates the *RRM2* gene expression level in patients aged less than or equal to 60 years. The blue rectangle box indicates the *RRM2* gene expression level in patients aged greater than 60 years. (**D**) The *RRM2* gene expression levels in different tumors belonging to different pathological stages. The red rectangle box indicates the *RRM2* gene expression level in stage I, the green one in stage II, the blue one in stage III, and the purple one in stage IV tumors. (**E**) Correlation analysis of RRM2 gene expression with OS, PFI, and DFI by the Cox regression analysis method in different types of cancers. * P < 0.05; ** P < 0.01; *** P < 0.001; **** P < 0.0001.

Analysis of the data obtained from TCGA and GTEx revealed that the expression level of RRM2 in the tumor tissues of ACC, BLCA, BRCA, CESC, CHOL, COAD, DLBC, ESCA, GBM, HNSC, KIRP, KIRC, LAML, LGG, LUAD, LIHC, LUSC, OV, PAAD, PRAD, READ, STAD, SKCM, THCA, TGCT, UCEC, THYM, UCS (P < 0.0001), SARC, and PCPG (P < 0.05) was higher than that of the control tissues (Figure 1B). However, no significant difference in RRM2 expression was observed between KICH and control tissues. Also, a higher expression of RRM2 total protein was observed in the primary tissues of BRCA, ovarian cancer, colon cancer, clear cell RCC, and UCEC. In this study, RRM2 expression data were generated for patients belonging to diverse age groups and tumor stages (Figure 1C, 1D). In BRCA, ESCA, KIRP, LUAD, LUSC, PCPG, PRAD, READ, and THYM tissues, the expression of RRM2 was higher in patients aged 60 years and above. Besides, we investigated the correlation of RRM2 expression with the prognosis of patients. For the TCGA cases, analysis of OS, progression-free interval (PFI), and disease-free interval (DFI) (Figure 1E) revealed a correlation between higher RRM2 expression and poor prognosis.

### Genomic alterations and methylation in RRM2

**Figure 2:**
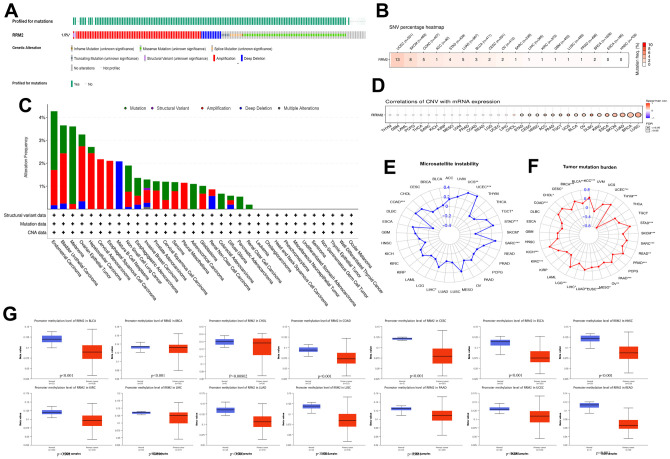
**Genetic alteration in *RRM2*.** (**A**) OncoPrint visual summary of alteration based on a query of RRM2. The green color represents mutation sites on a query of the *RRM2* gene. (**B**) The SNVs percentage profile of *RRM2*. The color in each small rectangle represents the mutation frequency in different types of cancers; the red and white indicate the high and low mutation frequency, respectively. (**C**) The alteration frequency of RRM2 with mutation type in different tumors. (**D**) CNV Pearson’s correlation between CNV and RRM2 mRNA expression. (**E**) Radar map of correlation between RRM2 expression and MSI. The value in black denotes the range, and the curve in red the correlation coefficient. (**F**) Radar map of correlation between RRM2 expression and TMB. The value in black reveals the range, and the curve in blue the correlation coefficient. (**G**) Analysis of promoter methylation levels of RRM2 in different tumors. *P < 0.05, **P < 0.01, ***P < 0.001.

**Figure 3:**
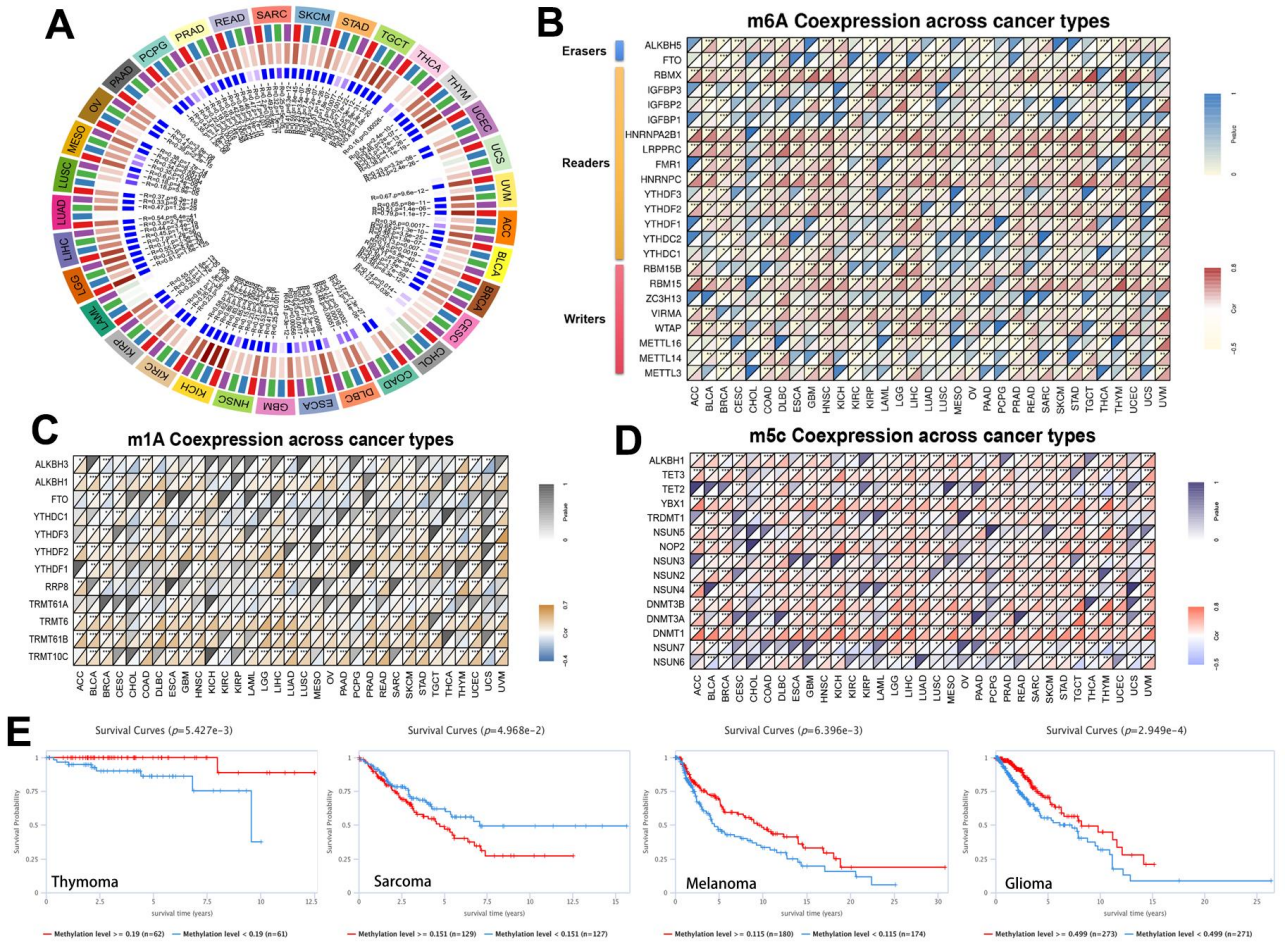
**Methylation in *RRM2*.** (**A**) Correlation between RRM2 expression level and DNA methyltransferases. DNMT1, red; DNMT2, blue; DNMT3A, green; and DNMT3B, purple. (**B**–**D**) Correlations between RRM2 expression and the m1A, m5C, m6A-related genes in different tumors. (**E**) Kaplan-Meier overall survival curves of different methylation levels of RRM2 in different tumors.

A total of 10,953 patients from the TCGA PanCancer Atlas Studies were obtained through the cBioPortal website. The percentage of *RRM2* genetic alterations was 1.5% (Figure 2A). The percentage of SNVs of the *RRM2* gene in UCEC and SKCM was 13% and 8%, respectively (Figure 2B). We performed a pan-cancer analysis of the genetic alteration status of *RRM2* and observed that maximum alteration (> 4%) appeared in patients with endometrial carcinoma (Figure 2C); patients with primary type of melanoma showed an alteration frequency of ~3%. The mutated site of RRM2 is depicted in the schematic diagram of the protein structure or the three-dimensional (3D) structure (Supplementary Figure 1B). Thus, the results indicated a strong relationship between RRM2 expression and mutations in multiple tumors. Also, a significant correlation was observed between gene-level and CNV in LUSC, BRCA, SKCM, ESCA, KIRC, DLBC, OV, BLCA, UCS, TGCT, PAAD, ACC, HNSC, CESC, and STAD (Figure 2D). As shown in Figure 2E, a significant correlation was observed between *RRM2* gene expression and MSI in COAD, LIHC, SARC, SKCM, STAD, TGCT, UCEC and, UCS. Tumor mutational burden (TMB), as a quantifiable biomarker, reflected the number of mutations in cancer. Significant correlations were observed between *RRM2* expression and TMB in ACC, BLCA, BRCA, CESC, CHOL, COAD, KICH, KIRC, LGG, LIHC, LUAD, LUSC, MESO, OV, PAAD, PRAD, SARC, SKCM, STAD, THYM, and UCEC (Figure 2F). Also, we compared the RRM2 methylation of different types of tumor tissues with normal ones. Studies have indicated that DNA hypermethylation silences the tumor suppressor gene, dysregulating crucial pathways related to malignancy. Results indicated significant differences in the following types of cancers: BLCA, BRCA, CHOL, COAD, CESC, ESCA, HNSC, KIRC, LIHC, LUAD, LUSC, PAAD, UCEC, and READ (Figure 2G). Pan-cancer analysis of DNA methyltransferases (DNMT) revealed its significant correlation with RRM2 (Figure 3A). We found that RRM2 was significantly associated with m6A, m1A, and m5C related genes (Figure 3B–3D). Also, results indicated that overall survival of different methylation levels of RRM2 exhibited a significantly different in thymoma, sarcoma, melanoma, and glioma (Figure 3E).

### The miRNAs and transcription factors of RRM2

**Figure 4:**
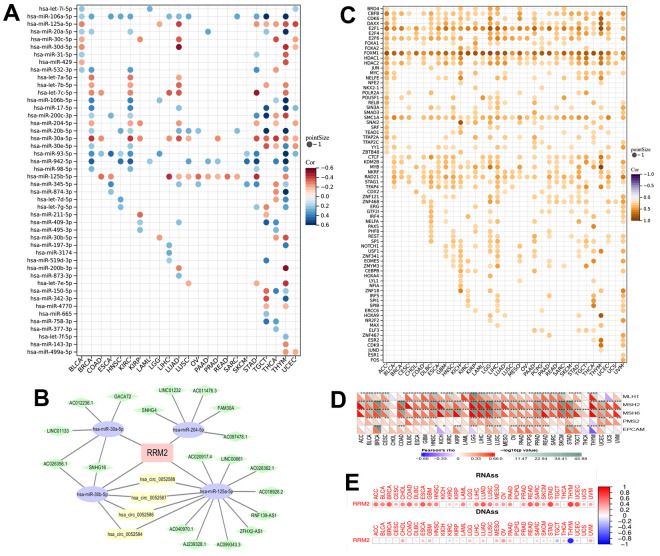
**The miRNAs and transcription factors of RRM2.** (**A**) The correlation of RRM2 expression with miRNA in different tumors. (**B**) The ceRNA diagram of lncRNA-miRNA-circRNA. The blue dots represent miRNA, green dots lncRNA, and yellow dots circRNA. (**C**) The transcription factor of RRM2 (p-value < 0.05 and correlation > 0.2). (**D**) Correlation between RRM2 expression and mismatch repair genes. (**E**) RRM2 gene expression associated with RNA stemness score (RNAss) and DNA stemness score (DNAss) in different cancers. Red dots indicate a positive correlation and blue dots a negative correlation. *P < 0.05, **P < 0.01, ***P < 0.001.

The correlation of RRM2 expression with miRNAs in different tumors is depicted in Figure 4A. A consistent negative correlation of has-miR-125b-5p and has-miR-30a-5p was observed in more than 10 types of cancers, indicating that these two miRNAs regulate RRM2 expression the most. Also, we constructed the ceRNA diagram of lncRNA-miRNA-circRNA in KIRC (Figure 4B) and predicted the transcription factors of RRM2 (Figure 4C). The following transcription factors were consistently positive in more than 20 cancer types and were predicted to be the potential targets of RRM2: CBFB, E2F1, E2F6, FOXM1, HDAC1, HDAC2, SMC1A, CTCF, and RAD21. We found that RRM2 was significantly associated with DNA mismatch repair (MMR) genes (Figure 4D). The expression of the *RRM2* gene also had a significant correlation with RNAss and DNAss in different cancers (Figure 4E).

### RRM2-associated biological functions and immune signatures

**Figure 5:**
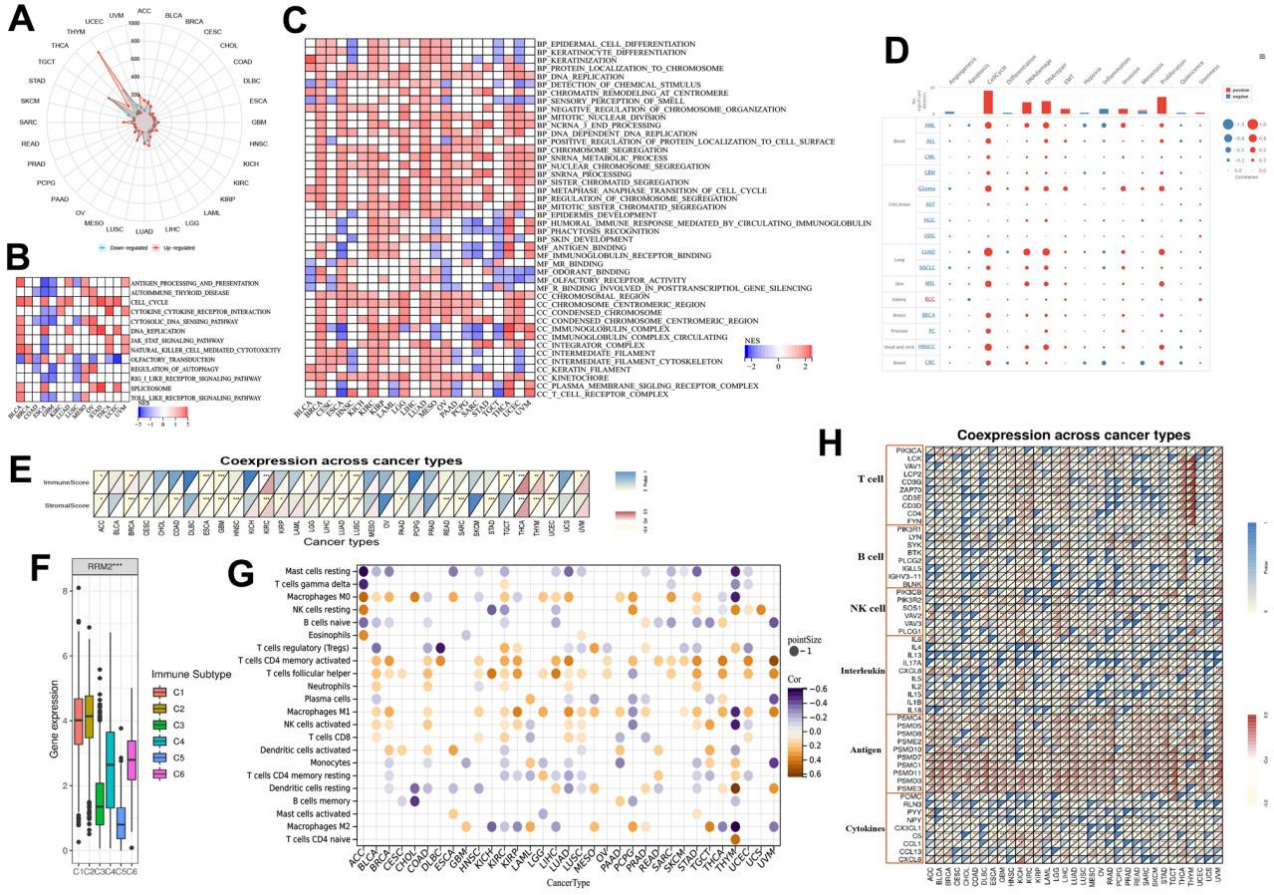
**RRM2-associated biological functions and immune signatures.** (**A**) The DEGs between the high- and low-RRM2 expression groups in different tumors. (**B**) The heatmap of gene KEGG analysis of DEGs. (**C**) The heatmap of GO analysis of DEGs between high- and low-RRM2 expression group. (**D**) Average correlations between RRM2 and functional status in different cancers and the bar chart indicating the number of datasets in which RRM2 is significantly related to the corresponding state for single-cell resolution. (**E**) *RRM2* gene expression associated with stromal and immune scores in different cancers. (**F**) *RRM2* gene expression levels in different immune subtypes. The X-axis represents the immune subtype; Y-axis gene expression. C1, wound healing; C2, IFN-g dominant; C3, inflammatory; C4, lymphocyte depleted; C5, immunologically quiet; C6, TGF-b dominant. (**G**) Correlation of *RRM2* gene expression with immune cell infiltration levels in 33 types of tumors (p < 0.05). (**H**) Correlation of *RRM2* gene expression with immune factors across human cancers. *: P-value <= 0.05; FDR <= 0.05.

To determine the biological functions of RRM2, we divided the tumor samples into high and low expression groups (Figure 5A). GSEA analysis results revealed that cell cycle, DNA replication, spliceosome, chromatin remodeling at the centromere, negative regulation of chromosome organization, mitotic nuclear division, ncRNA 3 end processing, DNA dependent DNA replication, chromosome segregation, snRNA metabolic process, nuclear chromosome segregation, snRNA processing, sister chromatid segregation, metaphase anaphase transition of the cell cycle, regulation of chromosome segregation, and mitotic sister chromatid segregation were positively associated with RRM2 in more than 10 types of cancers (Figure 5B, 5C). According to single-cell resolution results, the functions, including cell cycle, DNA damage, DNA repair, EMT, invasion, proliferation, and stemness were found to be positively associated with RRM2 (Figure 5D). Since accumulating evidence indicates that RRM2 regulates tumor immunity [10], we also paid attention to immune pathways. We observed that a substantial number of immune-related pathways had either positive or negative correlations with RRM2 depending on the cancer type (Figure 5B, 5C). A statistically significant relationship was observed between immune cell score and RRM2 expression in ACC, BRCA, ESCA, GBM, KIRC, LGG, LUAD, LUSC, TGCT, THCA, THYM, UCEC, and UVM (Figure 5E). The correlation analysis results indicated a potential correlation between RRM2 levels and immune subtypes in KIRC, apart from demonstrating that RRM2 was expressed in C2 at the highest level (Figure 5F). By examining the correlations between RRM2 and abundance of tumor-infiltrating immune cells, including myeloid and lymphoid lineages, across 33 cancer types, we observed that the levels of M0 and M1 macrophages, activated CD4+ T cells, and T follicular helper cells were consistently positive in more than 10 types of cancers. Moreover, the levels of resting mast cells, naive B cells, and resting memory CD4+ T cells were negatively correlated in more than 10 types of cancers (Figure 5G). Furthermore, correlations between RRM2 expression and immune factors across human cancers are depicted in Figure 5H. Therefore, the results demonstrated that RRM2 expression was notably associated with cancer immunity, though the regulation of RRM2 could vary depending on the cancer type.

### Identification of RRM2-related genes in different cluster subgroups

**Figure 6:**
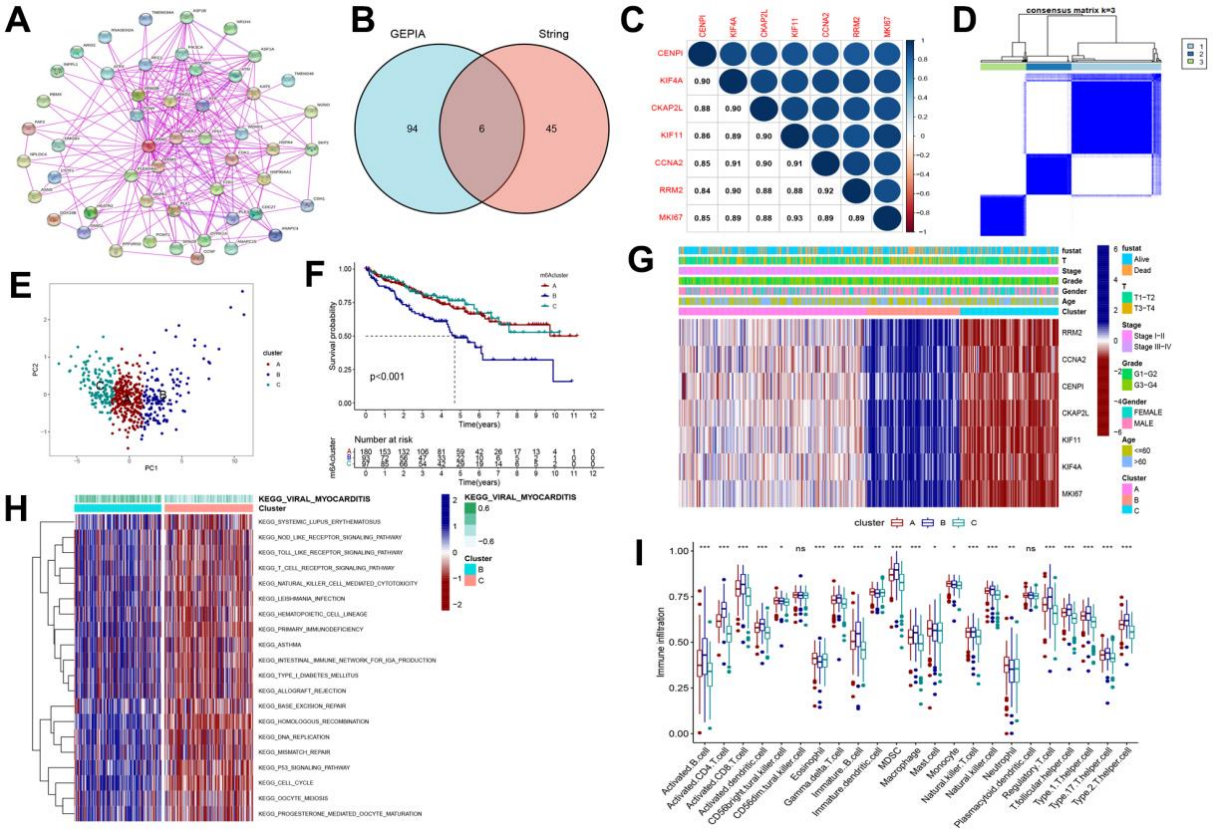
**Consensus clustering of RRM2-related genes in KIRC.** (**A**) Obtained the available experimentally determined RRM2-binding proteins from the STRING tool. (**B**) The intersection analysis of 100 genes from GEPIA2 and 50 genes from the STRING tool. (**C**) The correlation between the *RRM2* gene and RRM2-related genes. Blue dots indicate a positive correlation and red dots a negative correlation. (**D**) Consensus clustering matrix for k = 3. (**E**) Principal component analysis (PCA) for the transcriptome profiles of subtypes, indicating a significant difference in transcriptomes between different cluster subgroups. (**F**) Kaplan-Meier overall survival curves of different clusters. (**G**) Heatmap of three clusters defined by RRM2-related genes. (**H**) Differences in the Kyoto Encyclopedia of Genes (KEGG) pathways between cluster B and cluster C. (**I**) Comparison of immune infiltration level of primary RCC patients among three clusters, supported by single-sample gene set enrichment analysis algorithm. Data are presented as mean ±SD; ns P>0.05, *P<0.05, **P<0.01, ***P < 0.001.

To explore the potential mechanism of the *RRM2* gene, we screened *RRM2*-related genes and obtained a total of 50 RRM2-correlated genes from the String databases tool (Figure 6A). Next, we obtained the top 100 genes correlating with RRM2 expression based on the GEPIA2 tool. Finally, intersection analysis of the above two groups produced six common members, namely, CENPI, KIF4A, CKAP2L, KIF11, CCNA2, and MKI67 (Figure 6B); the heatmap demonstrated a strong positive correlation among them (Figure 6C). A total of 530 KIRC samples were classified into three clusters (262 patients labeled as “cluster A”, 133 patients labeled as “cluster B” and 135 patients labeled as “cluster C”) (Figure 6D, 6G). Results indicated that the RRM2-related genes were mostly expressed in cluster B (Figure 6E). Meanwhile, the survival analysis revealed that cluster B had a poor prognosis (Figure 6F). Furthermore, we analyzed DEGs between cluster B and cluster C subgroups and explored their functions through GSVA and KEGG pathway analysis (Figure 6H). Results indicated the enrichment of immune-related biological processes, including T cell receptor signaling pathway, natural killer cell-mediated cytotoxicity, primary immunodeficiency, the intestinal immune network for IgA production, and the P53 signaling pathway, involved in RRM2-related functions. Also, the differences in immune cell infiltration were observed in separate cluster subgroups (Figure 5).

### Establishing the risk score and evaluating the clinical predicting ability

**Figure 7:**
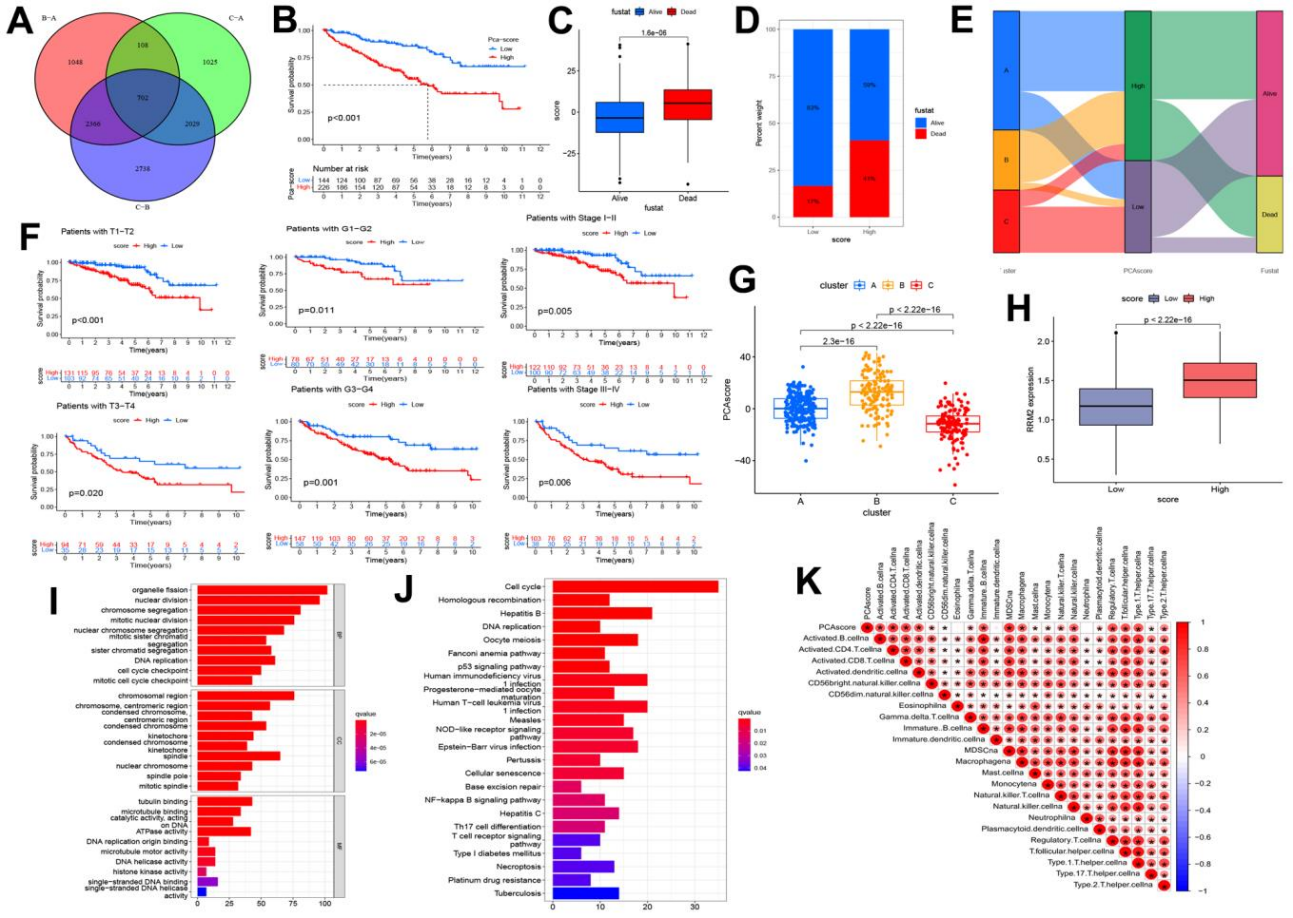
**Construction of the risk score and exploration of its clinical relevance.** (**A**) The Venn diagram of differential expression genes (DEGs) in three clusters. (**B**) Kaplan-Meier curves indicating the overall survival probability between two risk score groups. (**C**, **D**) Association of risk score with survival outcome. (**E**) Sankey diagram depicting the association of score groups with clusters, gene clusters, and survival outcome. (**F**) Kaplan-Meier curves demonstrating the overall survival probability in different clinical features. (**G**) The risk score difference between cluster A, cluster B, and cluster C. (**H**) High-and low-score groups are associated with the expression level of RRM2. (**I**) The GO analysis of prognostic DEGs. (**J**) KEGG analysis of prognostic DEGs. (**K**) The correlation between the risk score and immune cells.

The above analyses could not include other RRM2-co-expression genes and evaluate the biological function and significance of RRM2 comprehensively. Therefore, we constructed a risk score based on prognostic DEGs of different clusters (Figure 7A). Results indicated that patients exhibited a significantly shorter survival time in the high score group (Figure 7B–7D, 7F). The relationship between cluster and score is depicted in detail in Figure 6G, whereas the Sankey diagram indicated the connection between cluster, risk score, and outcome (Figure 7E). As we can see, cluster B was associated with the high-score group, which meant a poor prognosis. Also, we annotated the prognostic DEGs’ function (Figure 7I, 7J) and assessed that the risk score was positively correlated with immune infiltration levels (Figure 7K).

### Predicting immunotherapeutic resistance and related pathways

**Figure 8:**
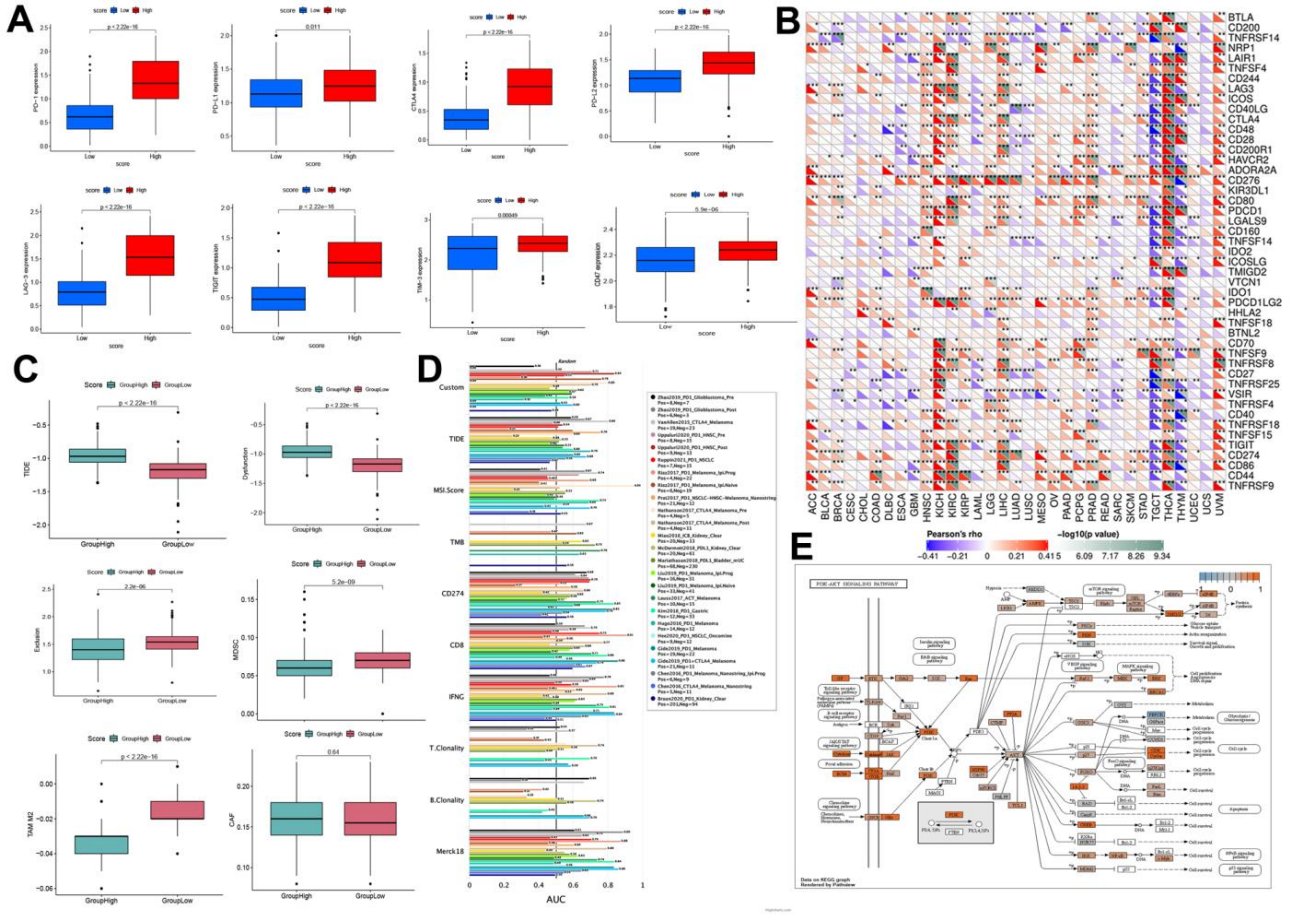
**Prediction of immunotherapy effect.** (**A**) High-and low-score groups associated with the expression levels of immune checkpoint molecules, including PD-1, CTLA-4, PD-L1, PD-L2, LAG-3, TIM-3, TIGIT, and CD47. (**B**) Relations between the expression of RRM2 and immunoinhibitors. (**C**) Relation of RRM2 expression with TIDE, dysfunction, exclusion, MDSC, TAM-M2, and CAF. (**D**) Comparison of the abilities of recognized biomarkers or genes in predicting RRM2’s response to immunotherapy. (**E**) Correlation of RRM2 expression with the PI3K-AKT signal pathway. P<0.05; ** P<0.01; *** P<0.001.

PD-1, PD-L1, PD-L2, CTLA-4, LAG-3, Tim-3, CD47, and TIGIT are immune checkpoint receptors widely used to evaluate the immune response. Results indicated that PD-1, CTLA-4, PD-L1, PD-L2, LAG-3, TIM-3, TIGIT, and CD47 receptors were significantly upregulated in the high-risk score group (Figure 8A). Moreover, we analyzed the relationship of immune checkpoint receptors with RRM2 expression in different types of cancers (Figure 8B). It was observed that RRM2 expression mainly correlated with immune checkpoint genes in tumor tissues of HNSC, KICH, KIRC, LIHC, PRAD, THCA, and UVM. Moreover, CD276 had a strong positive correlation with *RRM2* gene levels in 23 types of tumors. As we all know, tumor immunotherapy has emerged as an effective treatment for malignant tumors, and TIDE serves as a more accurate biomarker than immune checkpoint inhibitors (ICIs). In this study, the TIDE score, dysfunction, exclusion, MDSC, TAM-M2, and CAF were generated from the TIDE system. Results indicated that the risk score was positively correlated with the TIDE score, whereas dysfunction was negatively correlated with TAM-M2 and CAF (Figure 8C); this demonstrated that the high-score group had more side effects of ICIs, apart from having a poor survival prognosis. Meanwhile, a recent article reported that RRM2 overexpression reduced sunitinib sensitivity in renal cancer [10]. A comparison of the abilities of recognized biomarkers or genes in predicting RRM2’s response to immunotherapy is shown in Figure 8D. Based on the above results, we assumed that RRM2 was associated with ICI resistance. To explore the ICI resistance related signal pathways, the pathway map of RRM2 co-expression with PI3K-AKT signal pathway genes are shown in Figure 8E.

## DISCUSSION

Overexpression of RRM2 has been observed in various cancers, leading to its recognition as an effective cancer therapeutic target [11]. In this study, we performed a comprehensive bioinformatics-based analysis for RRM2 and built an RRM2-related risk score, which was a good predictor of survival outcomes and immunotherapy resistance.

High-expression levels of RRM2 can be regulated at gene, transcriptional, and post-transcriptional levels. Zhang et al. reported that the E2F1 transcription factor could upregulate RRM2 expression [12]. Besides, previous research has demonstrated that several transcription factors, such as FOXM1, MYC, APC/C/CDH1, and E2F, targeted RRM2 in prostate cancer [13]. In this study, we evaluated the correlation of all RRM2-related transcription factors with RRM2 in 33 types of cancers. Results indicated that the CBFB, E2F1, E2F6, FOXM1, HDAC1, HDAC2, SMC1A, CTCF, and RAD21 transcription factors were consistently positive in more than 20 types of cancers and were predicted to be the potential targets of RRM2. Besides, most studies have demonstrated that miRNA directly acts on mRNA, thereby mediating the post-transcription. Studies have reported that miR-20a-5p, miR-let-7, and miR-211 could regulate RRM2 expression [6, 9]. Our study demonstrated the correlation of RRM2 expression with miRNAs in different tumors. It was observed that has-miR-125b-5p and has-miR-30a-5p were negatively expressed in more than 10 types of cancers. Also, we constructed the ceRNA diagram of lncRNA-miRNA-circRNA in KIRC (Figure 3B). The results indicated that DNAss was correlated with epigenetic features, and RNAss was reflective of gene expression. Besides, the stemness index was related to tumor pathology and immune microenvironment, thereby predicting the clinical outcome [14]. In this study, a significant correlation of the *RRM2* gene expression was observed with RNAss and DNAss, especially in THYM (Figure 3). Significant correlations were observed between the expression of RRM2 and MMR genes, DNMT, and m1A, m5C, m6A-related genes. Thus, our study provided significant insights into the potential role of RRM2 in tumor immunology.

Previous studies have provided the effects of gene overexpression in different types of malignancies. Overexpression of ncRNAs and RNA modification-mediated RRM2 has been associated with poor survival of malignant tumors. Analysis of 159 breast cancer patients revealed that the *RRM2* gene levels were significantly associated with poor OS and PFS [15]. We investigated the expression of RRM2 in 33 different cancers using independent datasets from TCGA and GTEx databases. Results indicated that RRM2 was highly expressed in bladder, breast, colorectal, gastric, liver, kidney, lung, lymphoma tumors, etc. Also, its high expression was associated with OS, DFS, and PFI in ACC, KICH, KIRC, KIRP, LGG, LIHC, LUAD MESO PAAD, and SARC in the TCGA project. Interestingly, high expression of the *RRM2* gene correlated with an improved prognosis in THYM (Figure 1). In this study, we constructed a risk score to quantify the RRM2 expression pattern in individual primary KIRC. Our results demonstrated a poor prognosis of the high score group. These findings may provide insights for further investigation of the *RRM2* gene as a potential prognostic target.

Studies have indicated that the tumor microenvironment facilitates immunosuppression and limits immunotherapy responses [16]. In the last decade, immune infiltration, which might be a promising target in the tumor microenvironment, has gained attention in tumor progression and immunotherapy resistance [17]. We performed a pan-cancer analysis of the potential correlation between RRM2 gene expression and stromal score, immune score, tumor-infiltrating immune cells, and immune factors (Figure 4). Results indicated that the ncRNAs and RNA modification-mediated overexpression of RRM2 had a significant correlation with immune factors. We also identified three distinct patterns, which were correlated with different immune phenotypes and signaling pathways. Results indicated that KEGG pathways were negatively enriched in immune-related biological processes, including T cell receptor signaling pathway, natural killer cell-mediated cytotoxicity, primary immunodeficiency, the intestinal immune network for IgA production, and the P53 signaling pathway in the high-expression cluster of RRM2. Previous studies have demonstrated that ICIs are the main methods of immunotherapy [18]. In this study, further analyses revealed that PD-1, CTLA-4, PD-L1, PD-L2, LAG-3, TIM-3, TIGIT, and CD47 were significantly upregulated in the high-risk score group, implying that RRM2 could affect the efficacy of immunotherapy. Meanwhile, a key finding of this study was that patients in the low-score group exhibited a response to immunotherapy in KIRC. We discovered that the risk scores were positively correlated with the TIDE score and T cell dysfunction. The low-risk score group exhibited a better immunotherapy effect compared to the high-risk score group, indicating that a high-risk score group with high RRM2 expression might lead to the failure of ICI therapy. A similar result was reported in a study, wherein RRM2 overexpression reduced sunitinib sensitivity in renal cancer patients [10]. And we found RRM2 regulated the ICI resistance through the PI3K-AKT single pathways.

Our pan-cancer study employed comprehensive bioinformatic analyses for high expression of RRM2, demonstrating that it was related to the survival, prognosis, and effects of immunotherapy in cancer patients. Moreover, these findings may provide insights for further investigation of the *RRM2* gene as a biomarker in predicting immunotherapy’s response and therapeutic target.

## MATERIALS AND METHODS

### RRM2 expression and survival analyses in human cancers

The original data of 33 types of cancers were obtained from the public database TCGA and downloaded from the UCSCXenaShiny website (https://xena.ucsc.edu/). We searched RRM2 expression and retrieved data regarding overall survival (OS), progression-free interval (PFI), and disease-free interval (DFI) through the UCSCXenaShiny website [19]. Based on the Clinical Proteomic Tumor Analysis Consortium (CPTAC) dataset (http://ualcan.path.uab.edu/analysis-prot.html), we analyzed the differences in RRM2 protein level between normal and tumor tissue samples [20]. According to the clinical data of TCGA, the RRM2 expression levels among patients belonging to different age groups and pathological stages were analyzed.

### Genetic alterations and methylation in RRM2

The cBioPortal website (https://www.cbioportal.org/) was used to explore TCGA Pan-Cancer Atlas Studies of RRM2 [21]. Furthermore, the analysis of the association between RRM2 and tumor mutational burden (TMB) or microsatellite instability (MSI) was compiled using Spearman’s method. The single nucleotide variants (SNV) percentage profile and Pearson’s correlation on copy number variations (CNV) were performed based on the Gene Set Cancer Analysis (GSCA) website (http://bioinfo.life.hust.edu.cn/web/GSCALite/) [22]. The University of Alabama Cancer Database (UALCAN) (http://ualcan.path.uab.edu/) was utilized to analyze promoter methylation levels of RRM2 in different tumors [23]. Kaplan-Meier overall survival curves of different methylation levels of RRM2 in different tumors through the EWAS DataHub website (https://ngdc.cncb.ac.cn/ewas/datahub/index).

### ncRNAs and RNA modification of RRM2

The Encyclopedia of RNA Interactomes (ENCORI) (https://starbase.sysu.edu.cn/panCancer.php) was used to find the target-microRNAs (miRNAs) and long ncRNAs (lncRNAs) for RRM2. Co-expression analysis for the target-miRNA and RRM2 was performed through R package "Limma", “reshape2”, “ggpubr”, and “ggExtra” under corFilter < –0.2 or > 0.2 and p-valueFilter <0.05. Co-expression analysis for the target-miRNA and lncRNA was performed under corFilter <-0.1, p-valueFilter <0.05, and logFC* corFilter <0. Then RRM2 targeted-circRNA was obtained from the Circbank database (http://www.circbank.cn/index.html). Cytoscape served to construct the lncRNA-miRNA-circRNA ceRNA network for kidney renal clear cell carcinoma (KIRC). The transcription factors (TF) of RRM2 was analyzed through an online database (http://dbtoolkit.cistrome.org/), and the co-expression analysis for TF and RRM2 was performed through R package "Limma", “reshape2”, “ggpubr”, and “ggExtra” under corFilter > 0.2 and p-valueFilter <0.05. Using the SangerBox tools (http://www.sangerbox.com/tool), correlations between RRM2 expression and the m1A, m5C, m6A-related genes, and mismatch repair (MMR) genes were obtained. Moreover, the correlation analysis of RRM2, RNA stemness score (RNAss), and DNA stemness score (DNAss) was performed through R-package “Limma” and “corrplot”.

### RRM2-associated biological functions and immune signatures

To explore the RRM2-associated pathways, we performed Spearman’s correlation analysis for RRM2 expression and used Gene Set Enrichment Analysis to analyze the RRM2-associated biological functions. The C2 curated gene sets were downloaded from the Molecular Signatures Database (MSigDB). A gene ontology (GO) analysis was performed based on the false discovery rate (FDR) < 0.05 in more than 10 types of cancers. Kyoto encyclopedia of genes and genomes (KEGG) pathway analysis was performed based on FDR < 0.05 in more than three types of cancers. Average correlations between RRM2 and functional status in different cancers at single-cell resolution were performed through the CancerSEA database (http://biocc.hrbmu.edu.cn/CancerSEA/home.jsp) [24]. Stromal and immune cell scores were calculated by R package “estimate” and “Limma” [25]. Immune subtype analysis was performed utilizing R package “Limma”, “ggplot2”, and “reshape2”. Based on the Cell-type Identification by Estimating Relative Subsets of RNA Transcripts (CIBERSORT) method, the relative proportion of immune cell infiltrations in 33 types of cancers was evaluated [26].

### Identification of RRM2-related genes in different cluster subgroups

Based on the Search Tool for the Retrieval of Interacting Genes/Proteins (STRING) website (https://string-db.org/), a total of 50 RRM2-related proteins, supported by experimental evidence (low confidence [0.150]), were obtained. Also, the top 100 RRM2-correlated genes were obtained from the Gene Expression Profiling Interactive Analysis (GEPIA2) website. An intersection analysis was performed for the above two groups of RRM2-related genes. Correlation analysis between RRM2-related genes was performed by R-package “corrplot.” Next, the consensus cluster analysis, to classify the tumor samples into separate clusters built-in the expression profiles of RRM2-related genes in the KIRC TCGA datasets, was performed using the R package “ConsensusClusterPlus” [27]. The overall survival (OS) was calculated on the basis of the Kaplan-Meier method. R package Gene Set Enrichment Analysis “gsva” was used to find the KEGG enrichment score of the pathways [28]. Based on the R package “princomp”, principal component analysis (PCA) was carried out to validate the molecular subtype. The investigation on the immune infiltration landscape was performed based on the single-sample ssGSEA.

### Establishment of the risk score and evaluation of its clinical predicting ability

The differentially expressed genes (DEGs) were screened from different cluster subgroups using the R package “Limma”, and the prognostic DEGs were then selected for PCA. The following formula was utilized to establish the risk score = Σ(PC1i + PC2i), where “i” was the expression levels of the prognostic DEGs [29]. Kaplan-Meier curves, Sankey diagram, KEGG analysis, and the infiltration of the immune cells were performed by utilizing the R package “Limma”, “gsva”, and “ssGSEA”. The correlation analysis between the risk scores obtained for the expression of genes related to immune checkpoints, such as programmed cell death protein (PD)-1, cytotoxic T-lymphocyte antigen-4 (CTLA-4), programmed cell death ligand 1 (PD-L1), programmed cell death ligand 2 (PD-L2), lymphocyte activation gene-3 (LAG-3), T cell immunoglobulin and mucin domain-3 (TIM-3), T cell immunoreceptor with Ig and ITIM domains (TIGIT), and CD47, was done through R package “Limma.” The Tracking of Indels by Decomposition (TIDE) database (http://tide.dfci.harvard.edu/) was utilized for exploring the effect of RRM2 expression on immune dysfunction and immune exclusion [30]. Also, the effect of RRM2 expression on immune inhibitors was analyzed based on the SangerBox tool (http://www.sangerbox.com/tool). Based on the TCGA clinical data, the expression levels of the *RRM2* gene were analyzed by the treatment outcome of different cancer patients. Correlation between The Genomics of Drug Sensitivity in Cancer (GDSC) and RRM2-related genes was performed through the GSCALite database. The co-expression of RRM2 and the gene of resistance of immunotherapy-related signal pathway was performed based on the R package “Limma.” The heatmap was constructed based on the R package “reshape2” and “RColorBrewer.” Then, the liver hepatocellular carcinoma (LIHC) samples were subjected to “pathview” R package (p-value < 0.05) for the construction of pathway maps for PI3K-AKT signal pathway (hsa04151).

### Statistical analysis

In this study, the statistical operation and visualization were performed by utilizing the R 4.1.1 software. Based on the recommended methods, the statistical analysis for various data was performed based on different packages. Last but not the least, all tests were bilateral, and statistical significance was defined as P<0.05.

## Supplementary Material

Supplementary Figure 1
