# Supplementary material for: Comprehensive bioinformatics analysis of ribonucleoside diphosphate reductase subunit M2(RRM2) gene correlates with prognosis and tumor immunotherapy in pan-cancer
Source: Aging (Albany NY). 2022 Oct 3;14(19):7890–905. doi: 10.18632/aging.204315 (PMC9596216; doi:10.18632/aging.204315)
Supplement: Supplementary Figure 1 [file aging-14-204315-s001.pdf]

SUPPLEMENTARY FIGURE

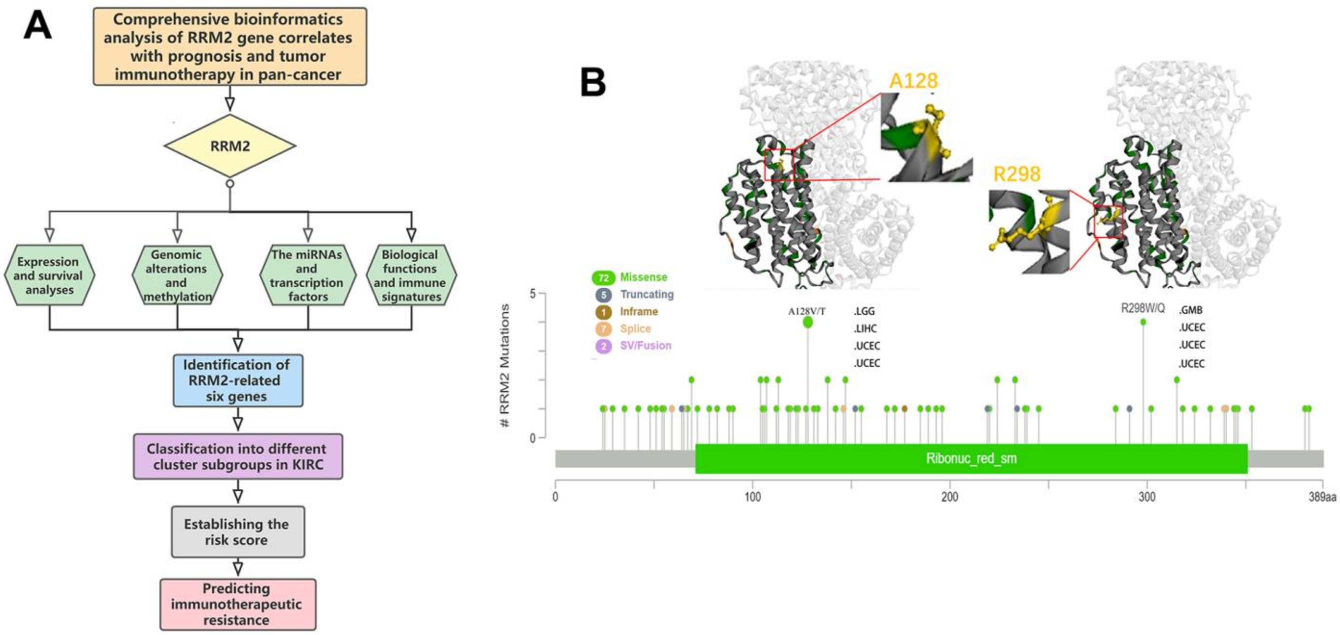

Supplementary Figure 1. (**A**) The overall study flowchart. (**B**) The mutated site of RRM2 is depicted in the schematic diagram of the protein structure or the three-dimensional (3D) structure.
